# Imaging flow cytometry reveals the mechanism of equine arteritis virus entry and internalization

**DOI:** 10.1038/s41598-025-87080-x

**Published:** 2025-01-25

**Authors:** Agata Kublicka, Daria Lorek, Agata Mikołajczyk-Martinez, Grzegorz Chodaczek, Aleksandra Chwirot, Barbara Bażanów, Anna Karolina Matczuk

**Affiliations:** 1 Department of Pathology, Division of Microbiology, Faculty of Veterinary Medicine, Wroclaw University of Environmental and Life Sciences, 50-375 Wroclaw, Poland; 2 Department of Immunology, Pathophysiology and Veterinary Preventive Medicine, Faculty of Veterinary Medicine, Wroclaw University of Environmental and Life Sciences, 50-375 Wroclaw, Poland; 3 Department of Biochemistry and Molecular Biology, Faculty of Veterinary Medicine, Wroclaw University of Environmental and Life Sciences, 50-375 Wroclaw, Poland; 4 Laboratory of Confocal Microscopy, Łukasiewicz Research Network – PORT Polish Center For Technology Development, Wroclaw, Poland

**Keywords:** Virus entry, Imaging flow cytometry, ImFC, Equine arteritis virus (EAV), Protease inhibitors, Virus-host interactions, Target identification

## Abstract

The process of viral entry into host cells is crucial for the establishment of infection and the determination of viral pathogenicity. A comprehensive understanding of entry pathways is fundamental for the development of novel therapeutic strategies. Standard techniques for investigating viral entry include confocal microscopy and flow cytometry, both of which provide complementary qualitative and quantitative data. Imaging flow cytometry, which integrates the advantages of both methodologies, offers significant potential in virological studies. In this investigation, we employed imaging flow cytometry coupled with immunostaining to monitor the entry of equine arteritis virus EAV into Vero cells via the endosomal trafficking route. Analysis provided an insight into the early infection dynamics across thousands of cells, revealing statistically significant alterations in internalization and uncoating process. Moreover, we evaluated the effectiveness of two inhibitors targeting cellular factors involved in facilitating viral entry: ammonium chloride, which disrupts endocytosis, and camostat mesylate, which inhibits the activity of serine proteases. The results demonstrated a clear distinction between effective and ineffective inhibitors. This study highlighted the potential of imaging flow cytometry to advance the study of viral entry and the evaluation of antiviral agents.

## Introduction

Overcoming cellular barriers is a critical step in establishing viral infection, making the improvement of research methods to visualize and quantify virus-host interactions essential for rapid diagnosis and the development of therapeutic candidates, such as antiviral drugs and monoclonal antibodies^1–3^.

The entry of a virus into a host cell is a complex process that involves subtle molecular changes on the cell surface or during the uptake of the virus. Single-virion tracking represents a highly effective method for investigating virus entry, offering real-time insights into entry kinetics and bridging the gap between structural and functional studies^4^. Detecting these events requires a precise tool with high sensitivity, as the initial stages of viral infection do not involve replication and thus do not follow the logarithmic growth phase typical for later stages^5^. Traditional experimental approaches that extend beyond the early phase of infection, capturing the eclipse phase or the full replication cycle, may not accurately reflect the true virus entry events. This is because these methods often measure a resultant of multiple, more complex processes rather than the direct entry of the virus into the cell. Consequently, there is a pressing need to refine techniques that offer much higher detection sensitivity and can accurately capture these early interactions^5^.

Bioanalytical methods traditionally used for studying virus entry include confocal microscopy and flow cytometry. Confocal microscopy is a powerful tool that enables the acquisition of optical sections to create high-resolution 3D images. This method facilitates imaging of early entry events from different cellular perspectives, thereby increasing sensitivity and precision compared to conventional microscopy techniques^6^. However, despite its many advantages, confocal microscopy is still primarily a qualitative method. Quantitative analysis of data, such as virion internalization, requires expensive software and manual image interpretation by humans, which can introduce bias. When applied to early viral events, it can be affected by artifacts, background noise, and a lack of quantitative homogeneity, which can complicate statistical analysis.

To address these limitations, flow cytometry is often employed to quantify and estimate the percentage of infected cells within an analyzed population. Originally intended for cytology, flow cytometry has become a versatile tool for analyzing various cellular processes, and it is particularly useful in virology due to the parasitic nature of viruses and their reliance on host metabolic functions. Flow cytometry is commonly used in diagnosis, evaluation of vaccine efficacy^7,8^, antiviral agents^9^, and research into host–pathogen interactions^2,10,11^. A critical step in both confocal microscopy and flow cytometry is the optimization of the immunostaining protocol, which must be tailored to the specific objectives of the experiment and the capabilities of the available research tools. Despite the strengths of these conventional techniques, both methods have limitations that can affect the accurate analysis of virus entry into host cells.

**Fig. 1:**
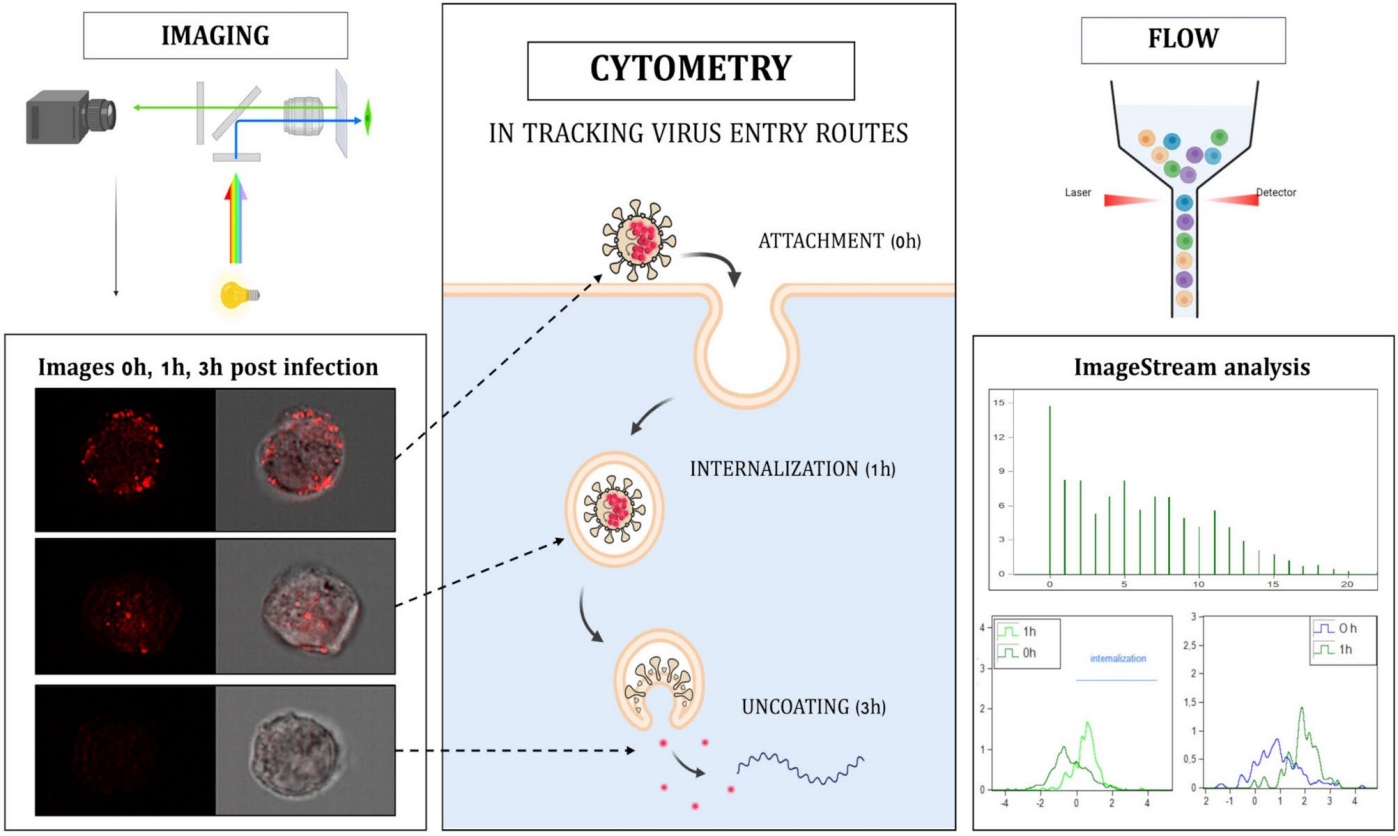
Imaging flow cytometry in tracking viral entry. Imaging flow cytometry allows visualisation of the early stages of viral infection by exploiting the optical capabilities of the instrument (left) in conjunction with simultaneous quantitative measurements (right). Images of cells fixed at different time points after infection (0 h, 1 h, 3 h) correspond to different stages of viral entry via endocytosis, including attachment, internalization and uncoating. In addition, parameters indicative of the extent of viral internalization during these early infection events were extracted through analysis using the IDEAS software.

Imaging flow cytometry (ImFC) offers a novel solution to these challenges by combining the strengths of both techniques (Fig. 1). ImFC provides simultaneous quantitative and qualitative measurements, capturing images of each cell while visualizing morphological and fluorescent signals^12,13^. This dual capability makes ImFC an invaluable tool in fields such as nanomedicine and microbiology, and it holds significant potential for virology research as well^14,15^.

In our study, we utilized equine arteritis virus (EAV), a prototype arterivirus assigned to the order *Nidovirales*, along with the family *Coronaviridae*^16^. EAV causes respiratory and reproductive disease in equids worldwide. The enveloped virions have diameters ranging from 50 to 74 nm, featuring glycoprotein-embedded envelopes and a nucleocapsid associated with RNA genetic material, which are consistent structural features characteristic for arteriviruses^16^. EAV is known to enter host cells via clathrin-dependent endocytosis^16–18^, but the precise mechanisms and the receptors involved in this process remain largely unknown. By leveraging the advanced capabilities of ImFC, we aim to gain deeper insights into the entry mechanism of EAV, thereby contributing to the understanding of viral pathogenesis and the development of targeted antiviral therapies.

## Results

### Visualization of EAV entry stages using confocal microscopy

To investigate the early stages of EAV entry (attachment, internalization, uncoating) into Vero cells we aimed to visualize the cells using confocal microscopy, following standard protocols for such analysis. Vero cells were infected with EAV at a high multiplicity of infection (MOI 50) to ensure robust visualization of the entry process. The infection was synchronized by incubating the virus with the cells at 4 °C for 1 h, allowing all virions to be at the same stage of entry.

**Fig. 2:**
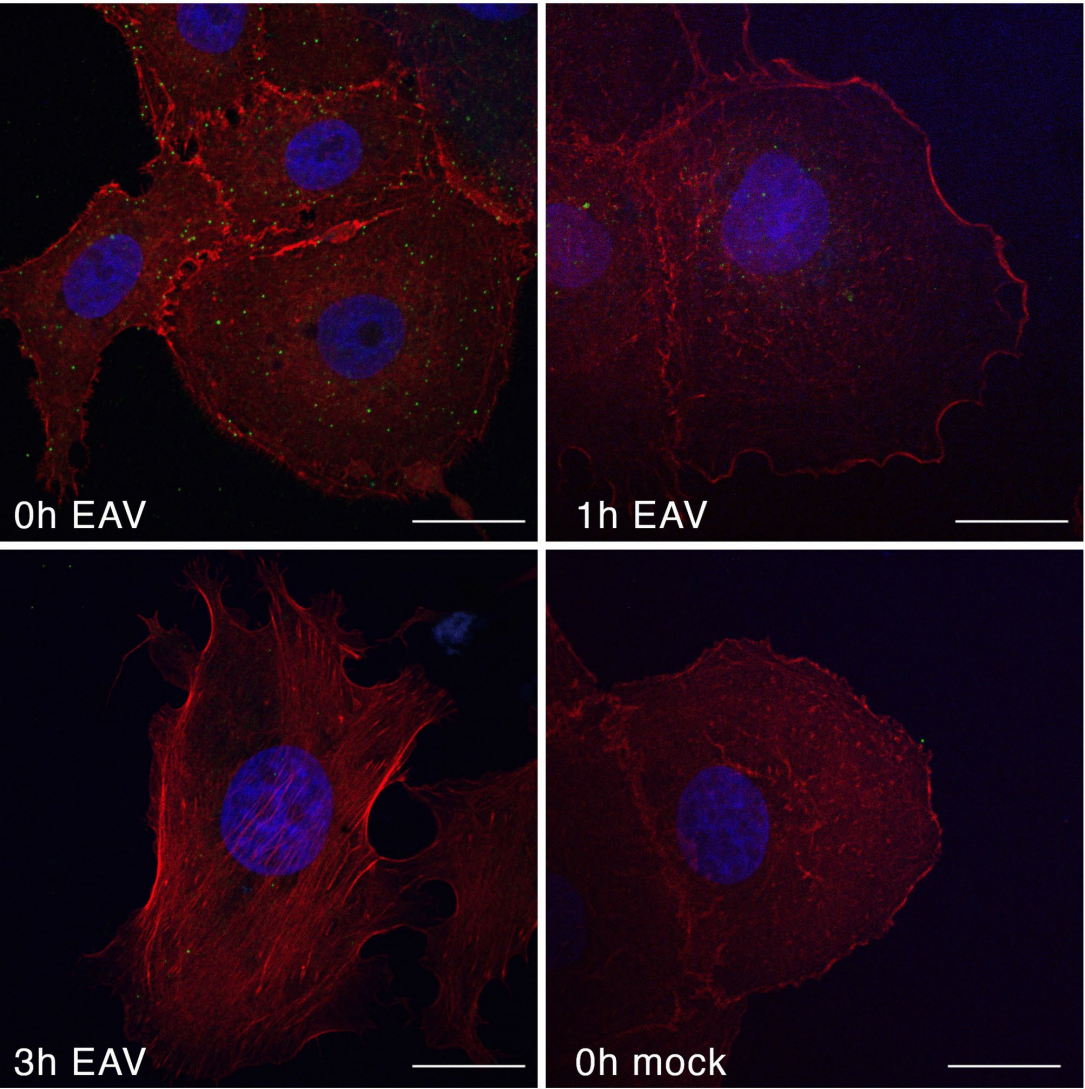
EAV entry into Vero cells visualised by confocal microscopy. Vero cells were synchronously infected with EAV or left uninfected. The cells were then fixed and subjected to immunostaining at different time points—0, 1 and 3 h post infection. An anti-N antibody was used to detect the viral nucleocapsid protein, allowing specific visualisation of the virus on or in the cells (green). To further visualise cell morphology and structure, actin filaments were stained with phalloidin (red) and nuclei with DAPI (blue). The pictures were taken with a confocal microscope (Zeiss Cell Observer SD, 63 × oil objective). Each scale bar represents 20 µm.

At time point 0 h, we observed that the virions were predominantly located on the surface of the cells, indicating the initial binding stage. By 1-h post-infection, most of the virions had been internalized, as evidenced by their localization within the cell. At the 3-h time point, no nucleocapsid signal was detected, suggesting that the entry process had been completed and the virions had fully uncoated within the host cells (Fig. 2).

These observations provide a clear temporal progression of EAV entry into Vero cells, from surface attachment to internalization and subsequent uncoating.

### Impact of cellular inhibitors on EAV infection cycle in Vero cells- conventional flow cytometry

**Fig. 3:**
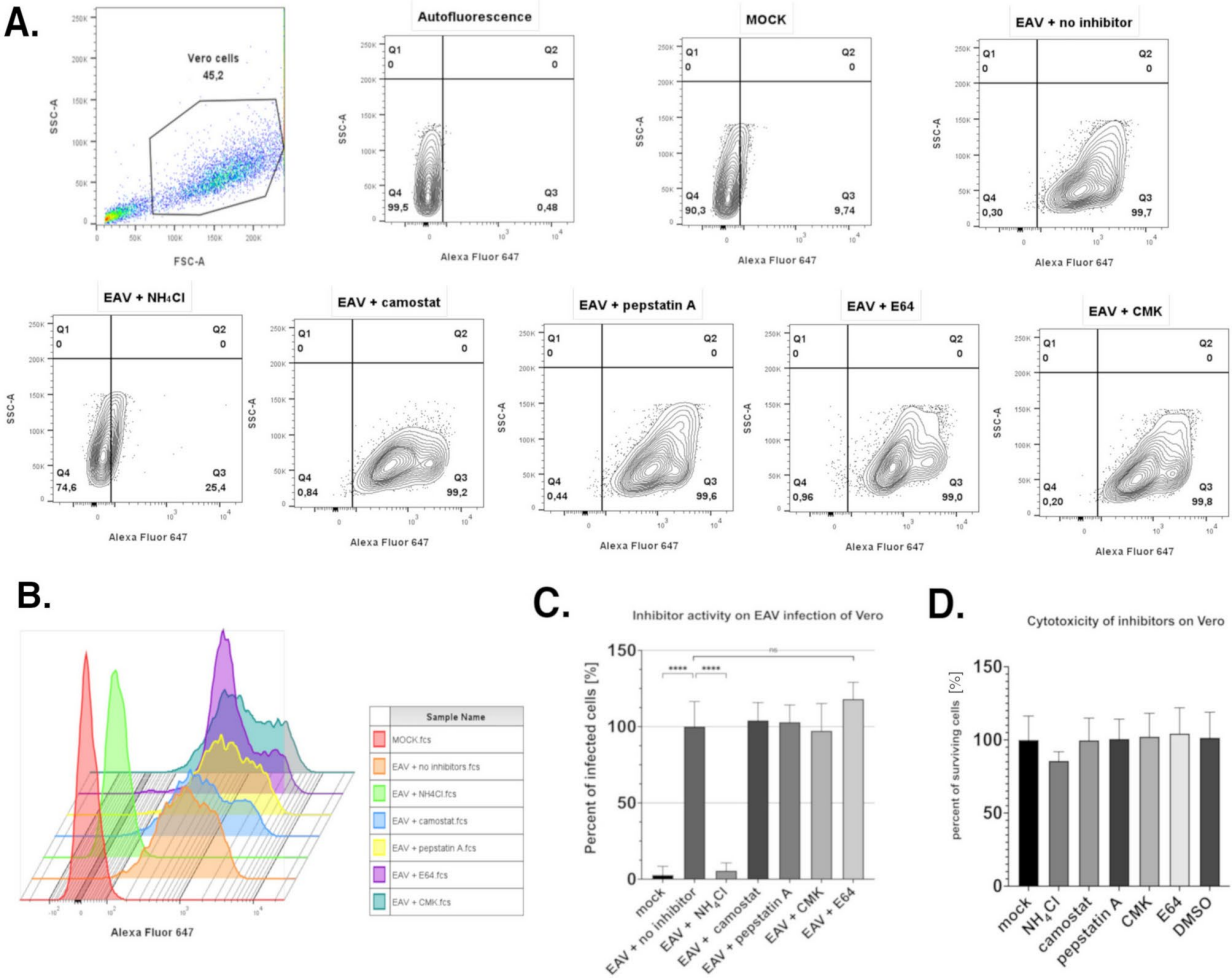
Impact of selected inhibitors on EAV replication in Vero cells. Vero cells were treated with the inhibitors and infected by EAV. The percentage of EAV positive cells were determined using conventional flow cytometry 36 h post infection. (**A**) Representative dot plot showing gating strategies for conventional flow cytometry. Vero cells were first gated to exclude debris and then the percentage of Alexa Fluor 647 positive (EAV infected) cells was determined. (**B**) Example overlay histograms from conventional flow cytometry showing fluorescence intensity in the Alexa Fluor 647 channel, reflecting virus concentration in Vero cells across different samples. (**C**) The graph presented the percentage of EAV-infected cells (36 h post infection) after treatment with selected inhibitors. The groups with inhibitors were each compared with infected control (EAV + no inhibitor). Statistical significance was detected between infected cells (EAV + no inhibitor) and mock (no infected cells) or EAV + NH_4_Cl (Alpha = 0,05, *p* < 0,0001) (****). In the case of the remaining inhibitors, no statistical significance was achieved (ns). (**D**) Cytotoxicity of inhibitors determined using the Cell Counting Kit-8 (CCK-8, TargetMol) and no statistically significant differences were observed compared with untreated control.

To investigate the role of pH and cellular proteases on the EAV infection we performed infection of Vero cells with various protease inhibitors and lysosomotropic agents. Our results show that only ammonium chloride (NH_4_Cl) impacts the EAV infectivity in Vero cells in contrast to other inhibitors that do not show statistically significant differences compared to cells infected with EAV without treatment (Fig. 3 A-C). The effects of those chemicals on EAV replication were measured with conventional flow cytometry 36 h after infection. The concentration of the inhibitors and chemicals were taken from the known acting concentrations on Vero cells and their cytotoxicity on cells was determined with the Cell Counting Kit-8 (Fig. 3D).

### Analysis of EAV entry using imaging flow cytometry

Because our experiment setting referred to the broad insight into infection cycle, it isn’t a precise model for study entry events. To develop a new method intended to entry events we performed ImFC.

To further quantify and visualize the entry of EAV into Vero cells, we conducted an experiment using imaging flow cytometry, which allows for simultaneous imaging and flow cytometric analysis of individual cells. The procedure mirrored the confocal microscopy experiment, with Vero cells being infected at a high multiplicity of infection (MOI 50) and the infection synchronized by incubating the virus with the cells at 4 °C for 1 h. After the infection, fixation of various time points (0 h,1 h,3 h p.i.) was performed, cells were permeabilized, blocked and then ImFC analysis was conducted.

**Fig. 4:**
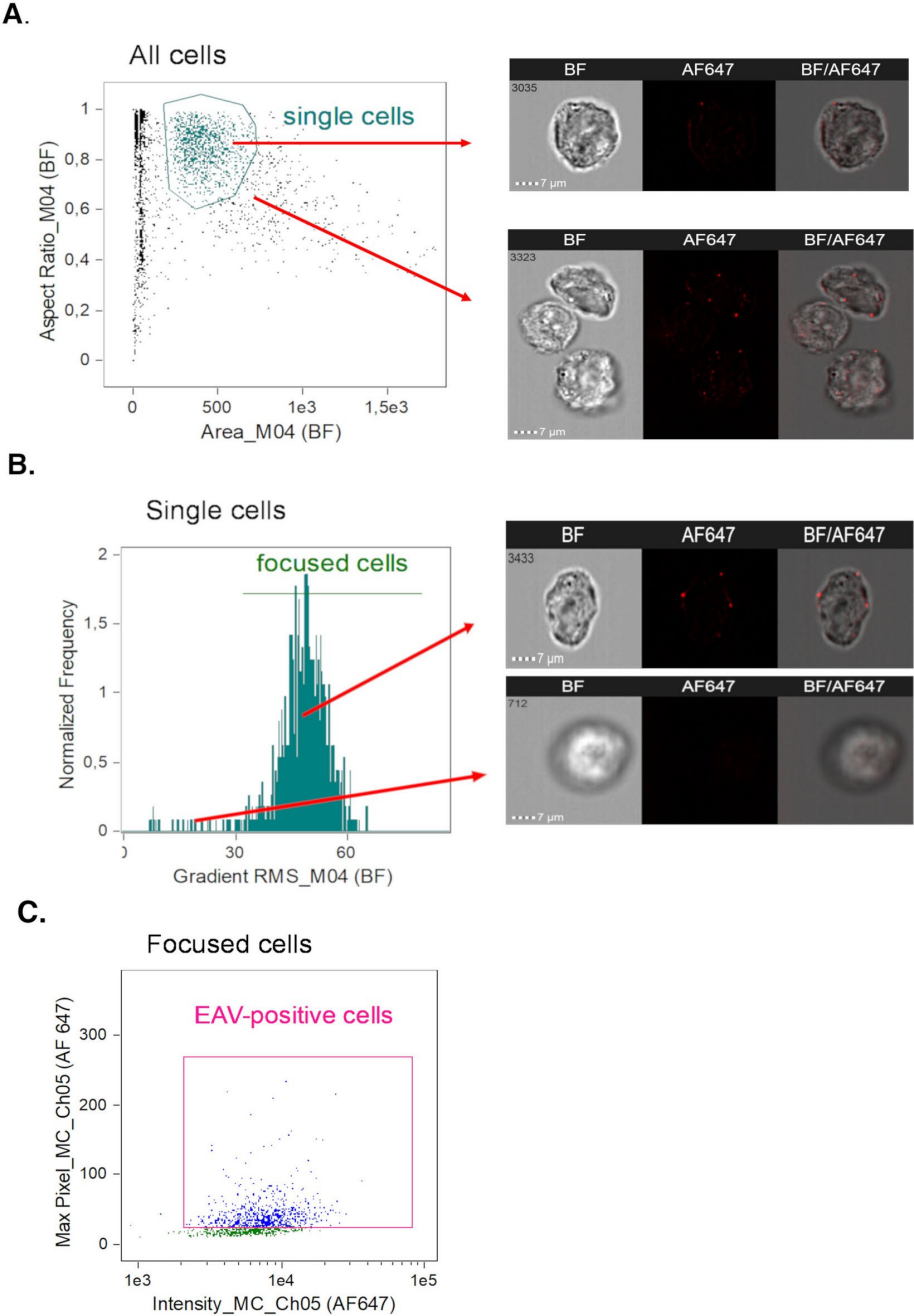
Schematic of the gating strategy with representative cell images for ImFC analysis.(**A**) Single Vero cells were gated from the total population using the Area and Aspect Ratio features on the BF images. (**B**) Focused cells were then selected using the Gradient RMS feature (BF channel). (**C**) Finally, Alexa Fluor 647-stained Vero cells (EAV-positive cells) were gated based on their Intensity and Max Pixel features.

The gating strategy was performed in order to select the population of separated, single cells (Fig. 4A), well-focused (Fig. 4B) and infected cells (Fig. 4C).

**Fig. 5:**
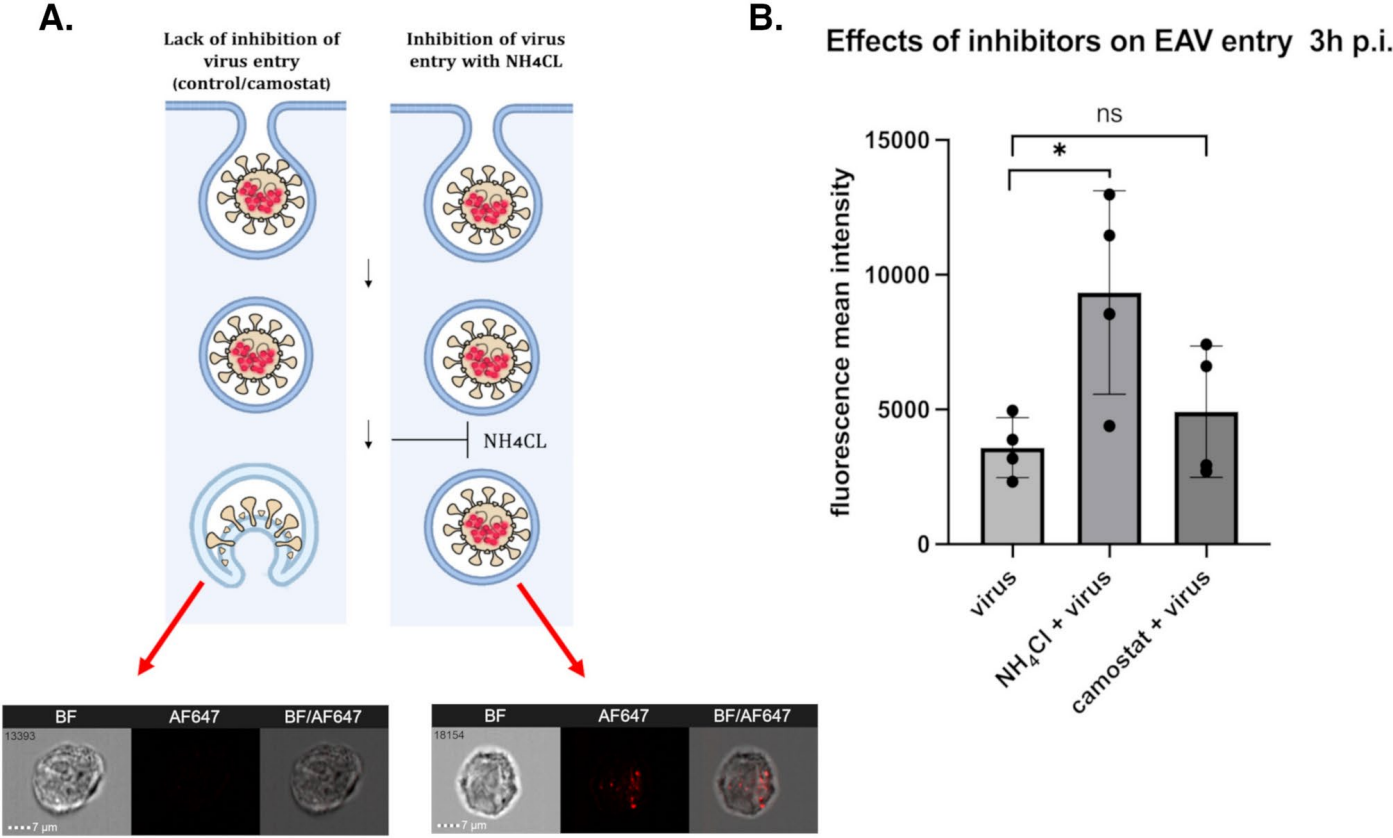
ImFC as a tool for differentiating effective from ineffective virus entry inhibitors. (**A**) A graphical representation illustrating the absence and presence of viral uncoating inhibition, with corresponding images from immunofluorescence microscopy (ImFC) taken 3 h post-infection. The cells were pre-treated with either NH_4_Cl or camostat, alongside a non-infected control, then fixed, permeabilized, blocked, and immunostained. ImFC analysis was conducted, and the captured images were presented as bright field (BF), AF647 fluorescence, and merged composites. In the control and camostat-treated cells, endocytosis is performed normally, and the fluorescent signal from the anti-N EAV antibody (red) is absent due to successful viral uncoating (left image). In contrast, NH_4_Cl -treated cells display a visible signal, indicating inhibition of endocytosis as a result of impaired acidification (right image). (**B**) The histogram highlights differences between positive and negative inhibition. The inhibition effect of NH_4_Cl was statistically significant (t-test, n = 4, p = 0.0265, two-tailed), while camostat showed no significant effect on uncoating (t-test, n = 4, p = 0.3586, two-tailed), when compared to the untreated control (infected cells without inhibitor).

Signal from the virions was detected on the cell surface as shown with merging brightfield and fluorescent channels at time 0. At time 1 h the signal was visible inside the cells. At 3 h the signal disappeared, it correlated with our results from confocal microscopy (Fig. 5 A).

Despite the consistent images obtained in the ImFC for the time points tested, the differences in signal intensity were not statistically significant, although the trend was clearly observed (Fig. 5B).

To measure virus uptake from the cell surface, we performed internalization measurements using the internalization feature of the Image Data Exploration and Analysis Software (IDEAS). This advanced capability allowed us to more accurately distinguish between surface-bound and internalized virions. Using this software, we observed a statistically significant difference in the internalization of EAV virions over time. At the 0-h time point, approximately 30% of the signal was classified as originating from inside the cells. By 1-h post-infection, around 70% of the signal was detected inside the cells, confirming a significant increase in virion internalization during this period (Fig. 5 C-D). Additionally, the software enables us to define a feature “spot count” which may reflect the number of virions per cell. Comparing time 0 h and 1 h we observed the statistically significant differences inversely to the internalization parameter (Fig. 5 E–F).

### The impact of inhibitors on the viral uncoating process- evaluation by ImFC

To investigate if the ImFC could be a useful method for testing active compounds directed at early stages of infection we selected one effective (NH_4_Cl) and one ineffective (camostat mesylate) inhibitor that affects EAV infection, as shown in our earlier experiment using conventional flow cytometry.

**Fig. 6:**
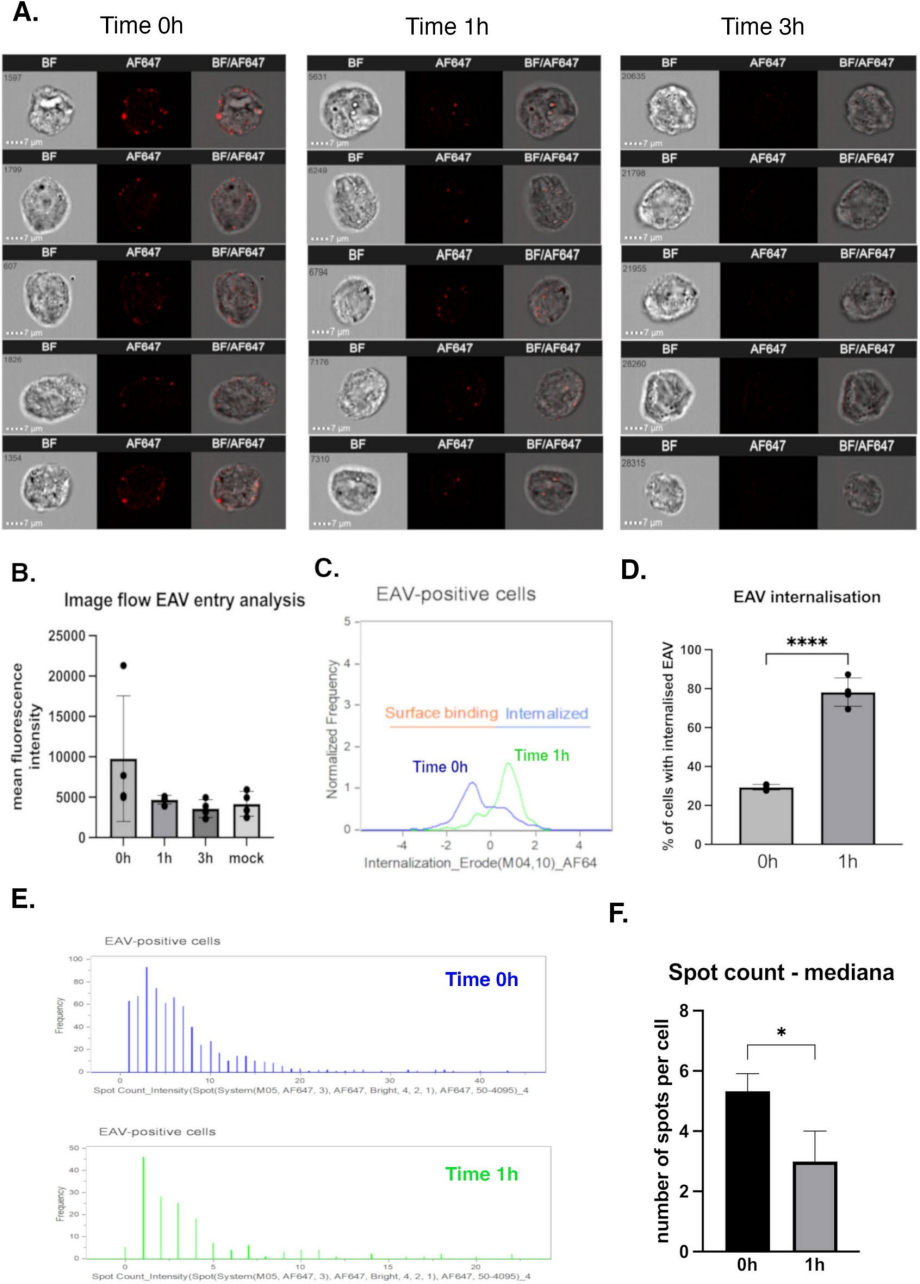
Investigation of equine arteritis virus (EAV) entry into Vero cells using imaging flow cytometry (ImFC). (**A**) The entry of equine arteritis virus (EAV) into Vero cells via endocytosis is illustrated by representative fluorescence images of infected cells at 0 h, 1 h, and 3 h post-infection. (**B**) Measurements of mean fluorescence intensity (MFI) showed a decreasing trend in fluorescence signals at later infection times (1 h and 3 h) compared to the initial time point (0 h). Mock-infected cells exhibited similar levels of fluorescence at 3 h. No statistical significance was observed. (**C-D**) Virus uptake, evaluated at 0 h and 1 h, revealed statistically significant differences in the percentage of cells with internalized EAV virions (t- test, n = 4, p < 0,0001, two- tailed). (**E-F**) The spot count parameter, calculated for the 0 h and 1 h stages, indicated a statistically significant difference in the number of virions per cell based on median (t-test, n = 3, p = 0,0249, two-tailed). At 0 h, more virions were detected (on the cell surface), while at 1 h, a lower number of viral particles were found (inside the cells). Bars and dots represent standard deviations and replicates, respectively.

Vero cells were pre-treated with NH_4_Cl, camostat, or left untreated as a control for 3 h. Following this treatment, the cells were submitted to synchronized infection with EAV, and after 3 h p.i. (a time point at which we had previously established that the signal from internalized virions is typically absent due to complete uncoating) with inhibitors were proceeded as earlier protocol and subjected to ImFC. (Fig. 6 A).

The results revealed that NH_4_Cl significantly inhibited the entry and/or uncoating of EAV, as evidenced by a statistically significant difference in mean fluorescent signal intensity compared to control cells (EAV infected, no inhibitor) (Fig. 6 B). In contrast, treatment with camostat, a serine protease inhibitor, did not show any significant effect on EAV entry into Vero cells. These findings confirm that while NH_4_Cl disrupts EAV entry, potentially by affecting endosomal acidification, camostat does not impact the virus’s ability to enter or uncoate within Vero cells (Fig. 6 B).

## Discussion

Our study aimed to explore the mechanisms of EAV entry into Vero cells and assess the role of cellular proteases by utilizing various protease inhibitors, along with imaging flow cytometry to evaluate its advantages and limitations in analyzing early viral entry steps. Our results demonstrate that the entry of EAV in Vero cells is solely pH dependent, which is consistent with previous findings by Nitschke et al. (2008), who showed that EAV enters BHK cells via clathrin-mediated endocytosis. Although our study did not specifically investigate whether EAV entry into Vero cells is clathrin-dependent, our observations using both confocal microscopy and imaging flow cytometry clearly indicate that virions are internalized into the cells, transitioning from the cell surface at the 0-h time point to being predominantly intracellular by 1-h post-infection. This internalization supports the notion of an endocytic entry mechanism.

Cellular proteases facilitate viral entry into cells by cleaving viral or host proteins, allowing conformational changes necessary for membrane fusion or uncoating of the viral genome. The exact mechanism of EAV entry and the potential role of proteases is not fully understood.

However, Nitschke et al. evaluated the effects of protease inhibitors such as E64 and leupeptin on EAV and found no inhibitory effects. In our study, we extended this investigation by testing a broader panel of protease inhibitors, including pepstatin A, camostat, and others. Similar to Nitschke’s findings, we observed no significant effect of these inhibitors on EAV replication in Vero cells. Notably, Nitschke used E64 at a relatively low concentration of 10 µM, while we tested at a higher concentration of 50 µM yet also did not observe any inhibitory effect. This suggests that cellular proteases might not play a crucial role in the EAV replication process in Vero cells.

Interestingly, when comparing our results with studies on other arteriviruses, such as porcine reproductive and respiratory syndrome virus (PRRSV), we find notable differences. Hou et al. (2020) demonstrated that pepstatin A, a pan-aspartic protease inhibitor, effectively inhibited the entry of PRRSV into MARC-145 (monkey kidney epithelial cells), a cell line permissive to PRRSV replication^19^. In contrast, our study did not observe any inhibitory effect of pepstatin A on EAV replication in Vero cells. This discrepancy could be attributed to differences in viral entry mechanisms between PRRSV and EAV or potentially to cell-type specific factors influencing the activity of protease inhibitors.

The entry mechanism of SARS-CoV-2 differs between Vero cells, which rely predominantly on endosomal cathepsin-mediated pathways, and other cell lines such as Calu-3 or Caco-2 cells, which use TMPRSS2-dependent plasma membrane fusion for viral entry^20^.

To investigate whether EAV employs an alternative entry pathway similar to SARS-CoV-2, we conducted an experiment using camostat mesylate in Vero cells. SARS-CoV-2, an enveloped virus from the order *Nidovirales*, utilizes several host cellular factors, including proteases, to facilitate viral entry by mediating the proteolytic cleavage and activation of the spike protein^21^. Proteases such as the serine protease TMPRSS2 (which can be blocked by camostat), cysteine cathepsins B and L, and furin enable the virus to overcome host barriers via different pathways^22^. We did not observe a comparable inhibitory effect of camostat on EAV infection, suggesting that serine proteases do not play a role in EAV entry in the Vero cell line.

A previous study of EAV entry was performed exclusively in BHK-21 cells^17^. These cells are commonly used because the virus achieves a higher titer when propagated in them compared to other continuous cell lines such as RK-13 and Vero. However, EAV has a broader cellular tropism than other arteriviruses such as PRRSV and SHFV, which replicate primarily in differentiated macrophages and certain macrophage-derived cell lines^23^. In the future, EAV entry into primary equine cell lines should also be evaluated.

In summary, it is likely that during the infection cycle, physical factors such as pH changes play a critical role in the EAV entry strategy. Our investigation of the effect of NH4Cl, which disrupts endosomal acidification, confirms that this compound significantly inhibits EAV replication (36 h p.i.) in Vero cells. This finding reinforces the idea that EAV relies on an acidification step within endosomes for successful entry and uncoating, suggesting that the endocytic pathway characterised by Nitschke et al. in BHK cells is likely to be operative in Vero cells. Acidification may be essential for inducing conformational changes in the EAV viral spike, particularly within the GP2/3/4 complex, rather than facilitating cathepsin activation through pH modulation. However, studies of fusion mechanisms in arteriviruses may be challenged by the lack of experimental or predicted structure (with alpha-fold ) for the spike protein, due to its inherent complexity^24^.

Studies using synchronized infection and pH-sensitive fusion assays suggest that the fusion of flaviviruses such as Dengue, Zika and West Nile viruses occurs within 15 to 30 min of infection in a variety of mammalian cell lines. This pH dependence was confirmed for Zika virus in the study by K. Owczarek et al. where compounds known to affect the fusion process, such as bafilomycin A and NH4Cl, inhibited envelope fusion with the target membrane at the late endosome stage, further suggesting that serine and cysteine proteases do not play a role in the virus entry^25^.

Traditional assays do not provide direct insights into the effects of inhibitors on the viral entry stage, which prompted us to develop and evaluate a novel imaging flow cytometry method. Our investigation into the entry of equine arteritis virus EAV into host cells revealed that complete uncoating occurs within a three-hour timeframe, with a notable decline in signal intensity evident after just one-hour post-infection. This reduction may suggest incomplete internalization—which we estimated to be around 70% or indicate that uncoating may begin prior to the one-hour mark. These observations imply that EAV fusion and uncoating are relatively rapid processes. Comparatively, previous studies concerning the porcine reproductive and respiratory syndrome virus (PRRSV) estimated that membrane fusion occurs approximately 45 min post-infection^19^, supporting the possibility that EAV may exhibit similar fusion dynamics.

In contrast, research on the influenza A virus (IAV) illustrates a slower progression, with peak colocalization with early endosomes occurring at 45 min and late endosomes at 120 min in A549 cells^26^. This discrepancy highlights the varying kinetics of fusion and uncoating among different viruses.

In our ImFC experiments, we did not observe statistically significant differences in overall mean fluorescence intensity between virus-infected and mock-infected cells, nor across the time points of 0, 1, and 3 h. Nonetheless, a trend emerged, indicating the highest signal intensity at time 0, followed by reductions at 1 h and further decline at 3 h for both the infected and mock-infected samples. Although these results were somewhat unsatisfactory, they highlight the challenges posed by the small size of virions and the nature of flow cytometry, which assesses the average signal across entire cell populations. High fluorescence spots detected may represent only a limited fraction of the total cell volume. Meanwhile, fluorescent images of cells highlighted distinct punctate staining patterns at the 0 and 1-h marks, with no detectable signal by 3 h. Unfortunately, the current software available for our cytometer does not enable quantification of the fluorescence signals of the dots captured in these images. Therefore, the software should be enhanced to include the analysis of high-intensity fluorescent points, representing virions, through the use of an automatic mask. This improvement would allow for the accurate counting of virions in each passing and imaged cell, thereby ensuring more precise data collection.

Besides the above-mentioned limitations, the current software allows quantification of virion internalization. Our measurements indicated that approximately 70% of the virus was internalized within one hour post-infection. In other studies using imaging flow cytometry and IDEAS software, the process of adenovirus internalisation in the A549 cell line was observed as early as 5 min after infection, reaching 50% and exceeding 95% after one hour^27^.

Post-flow cytometry image analysis, although semi-manual, was achievable within a few hours, demonstrating a time-efficient quantification method compared to conventional approaches for studying viral internalization. Historically, methods to study virion internalization, have required extensive time and resources, such as fluorescently labeling virions with proteins or tags, which is labor-intensive and complicated by the necessity of reverse genetics and stable cell line generation (e.g., for murine hepatitis virus)^28^. Furthermore, other microscopy techniques often rely on ultracentrifugation and require extensive protocol optimization^5^.

The ImFC method is simpler for analyzing early entry steps, allowing for analysis of wild-type viruses or newly emerging strains in unmodified cell lines. A limitation of our approach is the reliance on antibodies directed against structural viral proteins. The cost and availability of specific antibodies can pose challenges, particularly for emerging viruses or novel strains. Consequently, alternative methods such as RNA probes (FISH-cytometry), developed by Warren et al., have emerged as promising tools to study other arteriviruses, like the simian hemorrhagic fever virus. However, those assays measure cells approximately six hours post-infection, when viral replication is underway. Despite these limitations, FISH-cytometry remains a valuable approach for assessing the potential spillover of viruses from animal reservoirs to humans, as evidenced by its ability to demonstrate SHFV infection in human macrophages^29^.

In conclusion, our study successfully establishes ImFC as a novel method for assessing EAV entry and internalization dynamics, offering a time-saving and quantitative alternative to traditional techniques. However, it also emphasizes the need for software development intended for ImFC.

## Conclusions

We have successfully implemented imaging flow cytometry as a quantitative tool for studying virus entry, particularly in the context of inhibition and internalization studies. This method offers a more efficient approach to quantifying viral internalization compared to traditional techniques. Future improvements, particularly in software capabilities, could enhance the analysis of fluorescence signals derived from images rather than relying solely on flow cytometry data. Such advancements would provide a new perspective for the quantitative evaluation of viral entry processes and the efficacy of potential antiviral drugs. Overall, our findings contribute valuable insights into the dynamics of EAV infection and present image flow cytometry as a promising methodology for further virological research.

In this study, we demonstrate that complete uncoating of EAV in Vero cells occurs within three hours post-infection. Our findings indicate that cellular protease inhibitors, including pepstatin A, camostat, E64, and CMK, do not significantly impair EAV replication, as assessed by conventional flow cytometry. Conversely, the use of ammonium chloride (NH_4_Cl) revealed its importance in indicating pH-dependent entry mechanisms.

## Methods

### Cell lines

The Vero cell line (Vero, CCL-81™, ATCC, Manasaas, VA, USA) was maintained in Eagle’s Minimum Essential Medium (EMEM), supplemented with 10% heat-inactivated fetal bovine serum (FBS, Certified Fetal Bovine Serum), 100 U/ml penicillin, 0.1 mg/ml streptomycin (Penicillin–Streptomycin Solution), 100 mg/ml L-glutamine, and 1% non-essential amino acid solution.

The Baby Hamster Kidney (BHK-21, baby hamster kidney cells, C13, ATCC) cell line was cultured in a 1:1 mixture of Dulbecco’s Modified Eagle’s Medium (DMEM) and Leibovitz L-15 Medium supplemented with 5% fetal bovine serum, 100 U/ml penicillin, 0.1 mg/ml streptomycin, and 100 mg/ml L-glutamine.

All reagents used were obtained from Biological Industries, Kibbutz Beit- Haemek, Israel.

Both cell lines were incubated at 37 °C in an atmosphere containing 5% CO2.

### Virus propagation and quantification

Equine arteritis virus (EAV), strain Bucyrus (ATCC) was propagated in BHK-21 cell cultures. The virus titer was determined using a plaque assay performed on Vero cells. The plaque assay, which utilized carboxymethylcellulose as an overlay medium, was conducted to quantify the virus. After a 3-day incubation period, plaques were counted and PFU/ml was determined.

### Immunofluorescence assay and confocal microscopy

Vero cells were seeded onto 13 mm coverslips in a 24-well plate. The following day, the cells were infected with EAV at MOI of 50. Uninfected controls were incubated with supernatant collected from BHK-21 (prepared in parallel with EAV propagation). Adhesion was synchronized at 4 °C for 1 h. The cells were fixed using paraformaldehyde 4% in PBS (Thermo Fisher Scientific, Waltham, MA, USA) for 15 min at room temperature (RT) at 0, 1 h, and 3 h timepoints. After fixation, the cells were washed triply with phosphate-buffered saline PBS (Biological Industries). Then, they were permeabilized with 0.3% Triton (Sigma Aldrich, St. Louis, MO, USA) in PBS for 3 min at room temperature and washed again three times with PBS. The cells were then blocked with a solution containing 3% bovine serum albumin (Sigma Aldrich) in PBS for 1 h at room temperature. Next, primary antibodies specific to the N protein of EAV (VMRD, Pullman, WA, USA) were added to each experiment and incubated for 1 h at room temperature. Subsequently, the secondary antibodies, goat anti-rabbit IgG H&L Alexa Fluor 488, were applied at a dilution of 1:800 (Abcam, Cambridge, UK) simultaneously with Phalloidin iFluor 549 (Abcam) for F-actin staining at dilution 1:1000 for 1 h at room temperature. After the immunostaining process, cells were washed three times with PBS. Finally, the stained cell cultures were mounted onto glass slides using a mounting medium with DAPI (Abcam) and stored at 4 °C.

Images were captured using a Zeiss Cell Observer SD confocal microscope (Zeiss, Oberkochen, Germany) equipped with an EMCCD QImaging Rolera EM-C2 camera and 63 × oil objectives (0.167 and 0.106 μm per pixel, respectively). Imaging was performed sequentially using 405, 488, and 561 nm laser lines with a quadruple dichroic mirror 405 + 488 + 561 + 640 and 450/50, 520/35, and 600/52 emission filters.

### Inhibitors

Ammonium chloride (Sigma Aldrich) was dissolved in distilled water, camostat mesylate, E64, CMK. Pepstatin A was dissolved in DMSO. All used inhibitors were obtained from TargetMol, Boston, MA, USA. Inhibitors were filtered through a 0.22 µm syringe filter (Biokom, Poland).

### Cytotoxicity assay

Cell viability upon inhibitor treatment was tested using the ready-to-use Cell Counting Kit-8 (CCK-8, TargetMol), according to the manufacturer’s protocol. Briefly, cells were seeded in 96-well cell culture plates and subsequently treated with inhibitors for 6 h (for the virus entry test- imaging flow cytometry), 36 h (for the virus replication test- conventional flow cytometry) before incubation with 10 μl CCK-8 (1:10) for 1 h at 37 °C. After incubation, the absorbance at a wavelength of 450 nm was measured in a spectrophotometer microplate reader BioTek Epoch (Agilent, Santa Clara, CA, USA).

### Inhibitor treatment- pre-incubation

Cells were seeded in a 6-well plate at a density of 50,000 cells per well and incubated overnight at 37 °C. For testing impact of inhibitors on EAV infection cycle the Vero cells were pre-treated with concentrations of the compounds tested were respectively: ammonium chloride- 25 mM, camostat mesilate-10 µM, E64- 100 µM, CMK- 50 µM, pepstatin A -1 µg/ml. After the pre-treatment period, the medium containing the inhibitors was discarded, and equine arteritis virus (EAV) was introduced at a multiplicity of infection (MOI) of 50. The cells with the virus were incubated for 1 h at 4 °C to synchronize the infection without the presence of inhibitors. Following this, the virus was removed, the cells were rinsed with PBS, and the medium with inhibitors was replaced.

The cells were then incubated at 37 °C for 36 h.

### Conventional flow cytometry—sample preparation

Cells were fixed with 4% paraformaldehyde for 15 min, permeabilized with 0,3%Triton X-100 for 10 min, blocked in 3% BSA for 1 h. After each procedure cells were washed 3 times with PBS and centrifuged at 400 × g for 5 min. The following day the cells were immunostained via indirect method. Primarily the incubation with primary anti-N nucleocapsid protein-specific monoclonal antibody; equine arteritis virus EAV MAb IgG1 Isotype (VMRD) 1:250 in 1% BSA was performed for 30 min at room temperature, washed 3 times with PBS and centrifuged. Secondary Goat Anti-Mouse IgG Alexa Fluor 647 (Abcam) was added at concentration 1:2000 in 1% BSA and cells were incubated for 30 min, washed and centrifuged as previously. After the last wash cell pellet was resuspended in 500 µl of PBS. Cells were immediately proceeded up to 2 h.

### Conventional flow cytometry

The samples were analyzed using a FACSLyric™ flow cytometer (Becton Dickinson, Franklin Lakes, NJ, USA), and data analysis was performed using FlowJo v 10.8.1 software (Becton Dickinson). In the first step, forward versus side scatter (FSC vs SSC) gating was used to identify Vero cells and exclude debris from the analysis. Next, the percentage of infected cells was determined on the SSC vs Alexa Fluor 647 dot plot. The location on the axis of the EAV-negative population was determined based on the Alexa Fluor 647 fluorescence intensity of the no virus-staining control (autofluorescence).

### Imaging flow cytometry—sample preparation

After synchronization, cells were fixed with 4% paraformaldehyde on different time points 0 h, 1 h, 3 h post infection, then permeabilized with 100 µM digitonin (Sigma Aldrich) for 5 min in KHM buffer (110 mM potassium acetate, 2 mM MgCl_2_, 20 mM HEPES pH 7.2), blocked in 3% BSA overnight. After each procedure cells were washed 3 times with PBS containing 1% BSA, 1 mM EDTA and centrifuged at 400 × g for 5 min. The following day the cells were immunostained via indirect method. Primarily the incubation with primary anti-N nucleocapsid protein-specific monoclonal antibody; equine arteritis virus EAV MAb IgG1 Isotype (VMRD) 1:3000 was performed for 1,5 h at room temperature, washed 3 times with PBS containing 1% BSA, 1 mM EDTA, and 0,05% Tween20) and centrifuged. Secondary Goat Anti-Mouse IgG Alexa Fluor 647 (Abcam) was added at concentration 1:2000 and cells were incubated for 45 min, washed and centrifuged as previously. After the last wash cell pellet was resuspended in 20-30 µl of flow buffer (PBS without Mg2 + and Ca2 + , with 1 mM EDTA). Cells were immediately proceeded up to 2 h.

For the evaluation of effectiveness of inhibitors (uncoating) the Vero cells were pre-treated with 25 mM ammonium chloride (NH_4_Cl) and 10 µM camostat mesylate for 3 h. Both compounds were dissolved in EMEM supplemented with 10% FBS. After the pre-treatment period, the medium containing the inhibitors was discarded, and equine arteritis virus EAV was introduced at a multiplicity of infection (MOI) of 50. The cells with the virus were incubated for 1 h at 4 °C to synchronize the infection without the presence of inhibitors. Following this, the virus was removed, the cells were rinsed with PBS, and the medium with inhibitors was replaced.

The cells were then incubated at 37 °C for 3 h. Upon completion of the incubation period, cells were collected by trypsinization, washed, and centrifuged at 300 × g for 5 min and subjected to immunostaining as described above.

### Imaging flow cytometry analysis

The samples were acquired on an Image StreamX MK II (Cytek Biosciences, Fremont, CA, USA) imaging flow cytometer. Before each run, the flow cytometer was calibrated and tested using ASSIST, the Image Stream system’s calibration program. During acquisition, the 642 nm laser was set to 80 mW, the SSC laser at 785 nm was set to 1,88 mW, the magnification was set to a 60 × objective, and the fluidics were set to low speed. The Alexa Fluor 647 (AF647) signal was detected in channel Ch05, brightfield (BF) in channel Ch04, and SSC in channel Ch06.

Data analysis was performed using Image Data Exploration and Analysis Software Ideas® v6.2 (Cytek Biosciences). At least 1200 images and events were used for cellular analysis. In the first step, single-cell populations were identified by gating on cells with low brightfield area and high brightfield aspect ratio. Next, to determine the populations of cells in focus, the Gradient RMS (Root Mean Square) feature of the BF channel was used. The Gradient RMS feature measures the sharpness quality of an image by detecting large changes in pixel values and is commonly used to select focused objects when analyzing samples from an Image Stream imaging flow cytometer. Cells with Gradient RMS values > 32 was considered high-quality images.

Subsequently, we gated cells with higher Max Pixel values and Intensity values to identify only EAV-positive cells. This step also helped us exclude autofluorescence and nonspecifically stained cells (MOCK), which are characterized by lower intensity and Max Pixel values in the Ch05 (AF647) channel compared to virus-positive cells.

To evaluate internalized and surface-bound viruses, we used the Internalization feature, defined as the ratio of the intensity inside the cell to the intensity of the entire cell. The user has to create a mask to define the inside of the cell for this feature; therefore, we empirically created the following mask function, Erode (M04, 10) Ch05 (AF647), by customizing the default mask on the BF image (M04) by eroding it by 10 pixels. In our experiments, cells with a score > 0.3 were considered to have internalized particles, and those with a score < 0.3 were considered to have surface-bound viruses.

In turn, the number of viral particles attached to each cell (time 0 h) or inside the cells (time 1 h) was counted using the Spot Count feature. In this case, we used the following mask function: Spot Count_Intensity (Spot(System(M05, AF647, 3), AF647, Bright, 4, 2, 1), AF647, 50–4095)_4. First, we changed the system mask M05 from a weight of 5 to 3 to increase the likelihood of detecting the viruses in channel 5 (AF647). Then, we applied the Spot mask, setting the spot-to-cell background ratio to 4, the minimum radius to 1, and the maximum radius to 2. Finally, we added an intensity mask with a minimum intensity of 50 to cut off the background.

### Statistical analysis

Statistical analysis was performed using GraphPad Prism version 10. Analysis of Fig. 3 C-D was conducted by a one-way ANOVA. Analysis for Fig. 4B, Fig. 5 B, D, F was conducted by two-tailed, unpaired Student’s t-test. P-value for data sets, number of replicates indicated in the figure legends.

## Data Availability

Data supporting the findings of the study are available within the paper or from the corresponding author upon reasonable request.

## References

[1] Helenius, A. & Moss, B. Virus entry - An unwilling collaboration by the cell. *Curr. Opin. Virol.* **3**, 1–2. 10.1016/j.coviro.2013.01.003 (2013). 23395460 10.1016/j.coviro.2013.01.003 PMC4782761

[2] Mlelland, R. D., Culp, T. N. & Marchant, D. J. Imaging flow cytometry and confocal immunofluorescence microscopy of virus-host cell interactions. *Front. Cell. Infect. Microbiol.*10.3389/fcimb.2021.749039 (2021). 10.3389/fcimb.2021.749039 PMC8546218 34712624

[3] Jenner, D. C., Rieger, A. M. & Porat, Z. Editorial: The use of image and imaging flow cytometry as a tool to study host-pathogen interactions. *Front. Cell. Infect. Microbiol.*10.3389/fcimb.2022.1069960 (2022). 36467731 10.3389/fcimb.2022.1069960 PMC9714533

[4] Nathan, L. & Daniel, S. Single virion tracking microscopy for the study of virus entry processes in live cells and biomimetic platforms. *Adv. Exp. Med. Biol.* **1140**, 13–43 (2019). 10.1007/978-3-030-14741-9_2 PMC7122913 31317494

[5] Pohl, M. O. & Stertz, S. Measuring attachment and internalization of influenza a virus in A549 cells by flow cytometry. *J. Vis. Exp.*10.3791/53372 (2015). 26575457 10.3791/53372 PMC4692684

[6] Elliott, A. D. Confocal microscopy: Principles and modern practices. *Curr. Protoc. Cytom.*10.1002/cpcy.68 (2020). 31876974 10.1002/cpcy.68 PMC6961134

[7] De Rosa, S. C. Vaccine applications of flow cytometry. *Methods* **57**, 383–391 (2012). 22251671 10.1016/j.ymeth.2012.01.001 PMC3349786

[8] Gianchecchi, E. et al. Flow cytometry as an integrative method for the evaluation of vaccine immunogenicity: A validation approach. *Biochem. Biophys. Rep.* **34**, 101472 (2023). 37153861 10.1016/j.bbrep.2023.101472 PMC10160688

[9] Frezza, C. et al. Testing anti-HIV activity of antiretroviral agents in vitro using flow cytometry analysis of CEM-GFP cells infected with transfection-derived HIV-1 NL4-3. *J. Med. Virol.* **88**, 979–986 (2016). 26519867 10.1002/jmv.24418

[10] Notter, M. F., Leary, J. F. & Balduzzi, P. C. Adsorption of Rous sarcoma virus to genetically susceptible and resistant chicken cells studied by laser flow cytometry. *J. Virol.* **41**, 958–964 (1982). 6284984 10.1128/jvi.41.3.958-964.1982 PMC256832

[11] Van der Grein, S. G. et al. The encephalomyocarditis virus Leader promotes the release of virions inside extracellular vesicles via the induction of secretory autophagy. *Nat. Commun.*10.1038/s41467-022-31181-y (2022). 35750662 10.1038/s41467-022-31181-y PMC9232559

[12] Haridas, V., Ranjbar, S., Vorobjev, I. A., Goldfeld, A. E. & Barteneva, N. S. Imaging flow cytometry analysis of intracellular pathogens. *Methods* **112**, 91–104. 10.1016/j.ymeth.2016.09.007 (2017). 27642004 10.1016/j.ymeth.2016.09.007 PMC5857943

[13] Pirone, D. et al. Phenotyping neuroblastoma cells through intelligent scrutiny of stain-free biomarkers in holographic flow cytometry. *APL Bioeng*10.1063/5.0159399 (2023). 37753527 10.1063/5.0159399 PMC10519746

[14] Rees, P., Summers, H. D., Filby, A., Carpenter, A. E. & Doan, M. Imaging flow cytometry. *Nat. Rev. Methods Primers*10.1038/s43586-022-00167-x (2022). 37655209 10.1038/s43586-022-00167-x PMC10468826

[15] Dimitriadis, S. et al. Imaging flow cytometry: Development, present applications, and future challenges. *Methods and Protocols*10.3390/mps7020028 (2024). 38668136 10.3390/mps7020028 PMC11054958

[16] Brinton, M. A. et al. ICTV virus taxonomy profile: Arteriviridae 2021. *Journal of General Virology*10.1099/jgv.0.001632 (2021). 34356005 10.1099/jgv.0.001632 PMC8513641

[17] Nitschke, M. et al. Equine arteritis virus is delivered to an acidic compartment of host cells via clathrin-dependent endocytosis. *Virology* **377**, 248–254 (2008). 18570963 10.1016/j.virol.2008.04.041 PMC7103380

[18] Balasuriya, U. B. R., Go, Y. Y. & MacLachlan, N. J. Equine arteritis virus. *Vet Microbiol* **167**, 93–122 (2013). 23891306 10.1016/j.vetmic.2013.06.015 PMC7126873

[19] Hou, J. et al. Glycoprotein 5 Is cleaved by cathepsin E during porcine reproductive and respiratory syndrome virus membrane fusion. *J. Virol.*10.1128/JVI.00097-20 (2020). 32102888 10.1128/JVI.00097-20 PMC7199402

[20] Jackson, C. B., Farzan, M., Chen, B. & Choe, H. Mechanisms of SARS-CoV-2 entry into cells. *Nat. Rev. Mol. Cell Biol.* **23**, 3–20 (2022). 34611326 10.1038/s41580-021-00418-x PMC8491763

[21] Hoffmann, M. et al. SARS-CoV-2 cell entry depends on ACE2 and TMPRSS2 and is blocked by a clinically proven protease inhibitor. *Cell* **181**, 271–280 (2020). 32142651 10.1016/j.cell.2020.02.052 PMC7102627

[22] Oubahmane, M. et al. Host cell proteases mediating SARS-CoV-2 entry: An overview the international journal for in-depth reviews on current topics in medicinal chemistry. *Curr. Top. Med. Chem.* **22**(21), 1776–1792 (2022). 35894476 10.2174/1568026622666220726122339

[23] Meulenberg, J. J. & Snijder, E. J. The molecular biology of arteriviruses. *J. General Virol.* **79**(5), 961–979 (1998). 10.1099/0022-1317-79-5-961 9603311

[24] Matczuk, A. K., Kublicka, A., Chodaczek, G. & Siedlecka, M. Dual topology of equine arteritis virus GP3 protein and the role of arginine motif RXR in GP3 ER retention. *Virology* **597**, 110122 (2024). 38850896 10.1016/j.virol.2024.110122

[25] Owczarek, K. et al. Zika virus: mapping and reprogramming the entry. *Cell Commun. Signal.* **17**, 41 (2019). 31053158 10.1186/s12964-019-0349-z PMC6500006

[26] Edinger, T. O., Pohl, M. O., Yángüez, E. & Stertz, S. Cathepsin W is required for escape of influenza A virus from late endosomes. *mBio*10.1128/mBio.00297-15 (2015). 26060270 10.1128/mBio.00297-15 PMC4462628

[27] Herod, M. R., Pineda, R. G., Mautner, V. & Onion, D. Quantum dot labelling of adenovirus allows highly sensitive single cell flow and imaging cytometry. *Small* **11**, 797–803 (2015). 25285963 10.1002/smll.201401885

[28] Burkard, C. *et al.* Dissecting virus entry: Replication-independent analysis of virus binding, internalization, and penetration using minimal complementation of β-galactosidase. *PLoS One* **9**, (2014). 10.1371/journal.pone.0101762 PMC4099126 25025332

[29] Warren, C. J. et al. Quantification of virus-infected cells using RNA FISH-Flow. *STAR Protoc.* **4**(2), 102291 (2023). 37209094 10.1016/j.xpro.2023.102291 PMC10209735

